# Attenuating Neuronal Autophagy Alleviates Inflammatory Injury in OGDDeprived Co-culture of HT22 with BV2

**DOI:** 10.32607/actanaturae.11830

**Published:** 2023

**Authors:** Z. W. Huang, Y. Y. Liu, X. M. Chen, C. L. Yu, H. Y. He, Y. H. Deng

**Affiliations:** Department of basic medicine, Medical School, Kunming University of Science and Technology, Kunming, 650093 China; Anning First People’s Hospital Affiliated to Kunming University of Science and Technology, Kunming, 650093 China

**Keywords:** Ischemic stroke, neuronal autophagy, CX3CL1 expression, microglial inflammation, neuroprotection

## Abstract

Neuronal CX3CL1 suppressed microglial inflammation by binding to its receptor CX3CR1 expressed on microglia. Neuronal autophagy was prominently activated by cerebral ischemia, whereas CX3CL1 expression in autophagic neurons was conversely down-regulated to exacerbate microglial inflammation. Accordingly, this study was meant to investigate whether ischemia-activated microglial inflammation could be repressed by promoting CX3CL1 expression via the attenuation of neuronal autophagy. Immunofluorescence showed that autophagy predominantly occurred in neurons but barely in microglia. Western blot and immunofluorescence demonstrated that attenuating HT22 autophagy significantly increased its CX3CL1 expression and subsequently mitigated the BV2-mediated inflammatory responses, as indicated by decreased inflammatory factors of NF-κB-p65, IL-6, IL-1β, TNF-α, and PGE2. Meanwhile, CCK-8, Nissl staining, and FJC staining showed that an OGD (Oxygen-glycogen deprivation)-created neuronal injury was greatly alleviated by CX3CL1-suppressed microglial inflammation. Contrarily, elevating HT22 autophagy markedly decreased its CX3CL1 expression, which consequently worsened microglial inflammation and the neuronal injury. Our data suggests that attenuating neuronal autophagy may be an effective method to alleviate a microglial inflammatory injury after an ischemic stroke.

## INTRODUCTION

Cerebral stroke, a serious cerebrovascular disease, remains the main cause of disability and the second leading cause of death worldwide. Approximately 87% of patients suffer from an ischemic stroke [1]. The pathogenesis of cerebral ischemia has been investigated for decades, but means to alleviate post-stroke neurological injury remain troublingly few. A cascade of pathological processes causes neuronal death after an ischemic stroke, such as nutrient and energy depletion, release of reactive oxygen species, intracellular calcium overload, neuro-excitotoxicity, etc. [2]. Cerebral ischemia simultaneously activates a microglial inflammation and autophagic signaling. Microglia maintain cellular homeostasis by monitoring the microenvironment for responding to an injurious stimulus, such as ischemia. However, microglia-medicated inflammatory responses have been confirmed to be excessively amplified and, thereby, to accelerate the pathological aftershocks of an ischemic stroke [3]. A growing body of evidence demonstrates that autophagy remains prominently activated at the acute phase of a stroke. Yet this activated autophagy is predominantly displayed in neurons but seldom in microglia at the penumbra [4]. Recent studies have shown that there are close interactions between neuronal autophagy and a microglial inflammation [3]. Thus, understanding the mutual regulations between them might offer more clues for stroke treatment.

The chemokine fractalkine/CX3CL1 is a unique member of the CX3C family of chemokines. It is crucial in mediating the inflammatory response in the central nervous system [5]. Studies have revealed that communication between neurons and microglia is established via CX3CL1–CX3CR1 signaling [6]. CX3XL1 is only expressed on the membranes of neurons, while its receptor CX3CR1 is for the most part located on microglia [7]. Microglia are native inflammatory cells in the brain and are kept quiescent through conjugation with neurons by the CX3CL1–CX3CR1 contact under physiological conditions [8]. Thus, microglial activity is kept at an appropriate level to avoid triggering an excessive inflammatory response that can lead to neurological injury [9]. Thus, the CX3CL1– CX3CR1-mediated interaction between neurons and microglia was critical in maintaining normal brain function [10]. However, the inhibitory effects of neurons on the microglial inflammation are likely disrupted if the CX3CL1 and/or CX3CLR expressions are altered under a pathological state, such as cerebral ischemia [11]. Therefore, this study is meant to investigate what and how the CX3CL1-repressed microglial inflammation is disturbed, using an ischemia model of a co-culture of HT22 neurons with BV2 microglia.

Autophagy is a metabolic process by which damaged organelles, old proteins, superfluous cytoplasmic ingredients, and waste substrates to lysosomes are delivered for degradation [12]. At the same time, excessive autophagy accelerates cell death due to the uncontrolled autophagy initiation [13]. Autophagic/lysosomal signaling is prominently activated by cerebral ischemia [14]. Meanwhile, both reported studies and our previous investigations demonstrated that autophagy in neurons is excessively elevated by ischemic ischemia, leading to a massive accumulation of autophagic cargo within cells. Ultimately, the neurons at the penumbra suffer from autophagic cell death [15]. Intriguingly, more evidence shows that autophagy predominantly occurs in neurons, but seldom in microglia after an acute ischemic stroke [1]. Based on the CX3CL1–CX3CR1-mediated crosstalk mechanism, we asked ourselves whether the CX3CL1 expression could be changed in autophagic neurons, thereby subsequently weakening the suppressive effect of neurons on a microglial inflammation [5]. Consequently, the microglia-triggered inflammation response can be amplified to increase neuronal death [16]. To verify this hypothesis, a rat model of ischemic stroke was prepared to attempt to better understand the correlation between neuronal autophagy and microglial inflammation in our previous study [16]. The results showed that ischemia-induced neuronal autophagy leads to a reduction of the CX3CL1 expression. Moreover, further autophagy decreases the CX3CL1 expression and, consequently, aggravates the microglial inflammation and neurological injury. Conversely, attenuating autophagy significantly elevates the CX3CL1 expression of neurons, which in turn alleviates the microglial inflammatory injury and brain damage. These data support the contention that neuronal autophagy aggravates the microglial inflammatory injury by down-regulating the CX3CL1 expression on neurons. However, the study failed to elucidate the direct regulative mechanism of neuronal autophagy on a microglial inflammation after cerebral ischemia.

To investigate the direct crosstalk mechanism between neuronal autophagy and a microglial inflammation, an OGD co-culture of HT22 neurons with BV2 microglia was first prepared in this study. Thereafter, the culture condition was made to meet the requirement that autophagy is mostly induced in HT22, but rarely in BV2. Based on this understanding, the autophagy level in the neurons is pharmacologically altered to reveal the exact effect of neuronal autophagy on the microglial inflammatory response. The effect of a autophagy-regulated microglial inflammation on a neuronal injury is correspondingly explored. Through our study, the correlative regulation between neuronal autophagy and a microglial inflammation after cerebral ischemia ought to be fully elucidated.

## MATERIALS AND METHODS

**1.1. Cell culture**

Mouse hippocampal neuron (HT22) and mouse microglial cells (BV2) were purchased from Wuhan Procell Life Technology Co., Ltd (Wuhan, China). HT22 and BV2 cells were firstly cultured in a high glucose DMEM medium (Hyclone, UT, USA) containing 10% fetal bovine serum (Biological Industries, CT, USA), respectively. After 2 days of separated culture, the HT22 and BV2 cells were collected and counted, and they were seeded into T25 culture flasks at a ratio of 9 : 1 for co-culture. After 24 h of co-culture, the model of oxygen-glucose deprivation/reoxygenation (OGD/R) was ready.

**1.2. Oxygen-glycogen deprivation/reoxygenation (OGD/R)**

To prepare the model of cell ischemia *in vitro*, the complete co-culture medium of HT22 with BV2 was replaced with a serum-free, sugar-free medium (glucose deprivation). The culture plates were placed in 95% N2 and 5% CO_2_ chambers (oxygen deprivation). After 1.5 h of OGD, the culture medium was replaced with a complete DMEM medium (resupply of glucose) and the plates were moved into the incubator with 5% CO_2_ (reintroduction of oxygen). In this way, a OGD cell model mimicking the microenvironment in the ischemic brain tissues was created. The main objective of our study was to establish the correlation between neuronal autophagy and a microglial inflammation. For this reason, the culture conditions had to be adjusted to account for the fact that autophagy is mostly induced in neurons but little in microglia. Following this, the autophagy level in the HT22 and BV2 cells, respectively, was measured by double immunofluorescence.

We investigated how neuronal autophagy affects its CX3CL1 expression, which subsequently regulates the microglial inflammatory response by altering CX3CL1–CX3CR1 signaling. The autophagy inducer Tat-Beclin1 and inhibitor 3-methyladenine (3-MA, 15 μM) were additionally added into the co-culture medium. Our preliminary study had confirmed that a dose of 15 μM Tat-Beclin1 could further promote autophagy in HT22 cells upon OGD, whereas the same dose of Tat-Beclin1 had little effect on the autophagy level in BV2 in the co-culture.

**1.3. Western blot**

The total proteins of the co-cultured cells were extracted using a protein extraction kit (Beyotime Biotechnology, Shanghai, China). After quantification by the BCA method, the proteins were separated by molecular weight using a polyacrylamide gel before being transferred onto PVDF membranes (Millipore Corporation, Ma, USA). Nonspecific proteins were blocked with 10% nonfat milk for 2 h at room temperature. After washing with TBST, the PVDF membranes were incubated with rabbit primary antibodies against mouse LC3 (1 : 10000, Sigma, MO, USA), beclin1 (1 : 1000, ABclonal, Wuhan, China), CX3CL1 (1 : 2000, GeneTex, CA, USA), NF-κB-p65 (1 : 1000, GeneTex, CA, USA), and β-actin (1 : 10000, Abclonal, Wuhan, China) overnight at 4°C. After the washing step, the secondary antibodies were labeled for 2 h at room temperature. The fluorescence signal intensity was analyzed by Image J, and the band density values were normalized to β-actin.

**1.4. Immunofluorescence**

The co-cultured cells were seeded onto six-well plates coated with polylysine. Thereafter, the cells were permeabilized with 0.2% Triton-X100 for 5 min and washed with PBS. After blocking with 10% BSA (Beyotime Biotechnology, Shanghai, China) for 1 h at room temperature, the rabbit primary antibodies against rat LC3 (1 : 400, Sigma, MO, USA), NeuN (1 : 400, Abcam, Cambs, UK), Iba-1 (1 : 400, Abcam, Cambs, UK), CX3CL1 (1 : 400, GeneTex, Texas, USA), and NF-κB-p65 (1 : 400, GeneTex, TX, USA) were incubated overnight at 4°C. After washing, they were labeled with Alexa Fluor-coupled secondary antibodies (1 : 1000, Jackson ImmunoResearch Laboratories, INC. PA, USA) in the dark. Finally, the cells were washed and fixed with DAPI (1 : 2000, Sigma, MO, USA). The results were represented in the form of fluorescence intensity. Under high magnification (×200), the fluorescence density was calculated by Image J and the fluorescence density values required background removal.

**1.5. Enzyme-linked immunosorbent assay (ELISA)**

The HT22 and BV2 co-cultured cells were collected by centrifugation, and the culture medium was also obtained for the measurement of inflammatory factors. The concentrations of TNF-α, IL-6, IL-1β, and PGE2 in the co-culture medium were measured by an ELISA kit (Biotech & Jingmeibio, Beijing, China), according to the instructions provided by the manufacturer. The detected values were quantified according to the standard curve.

**1.6. Nissl staining**

The co-cultured cells seeded on the six-well plates were firstly fixed with 4% paraformaldehyde and then dehydrated by 70% ethanol for 1 min. Following that, they were immersed in a Cresyl violet Stain solution (Leagene, Beijing, China) at 56°C for 1 h and then rinsed with deionized water. Thereafter, the Nissl Differentiation solution (Leagene, Beijing, China) was added to incubate for 2 min. Finally, rapid dehydration using ethanol, as well as verification using xylene, was conducted. The staining was observed and photographed with a stereoscopic microscope (Nikon Instruments Co., Ltd., Tokyo, Japan). The result was expressed as the number of Nissl bodies in 10 randomly selected non-overlapping fields under high magnification (×200). Five plates had to be counted for each group.

**1.7. Fluoro-Jade C (FJC) staining**

A Fluoro-Jade C (FJC) Staining Kit (Thermofisher, MA, USA) was used to detect the necrotic neurons, according to the instructions provided by the manufacturer. The co-cultured cells were processed with sodium hydroxide from Solution A for 5 min and incubated with 70% ethanol for 2 min, then washed in distilled water for 2 min. The cells were further incubated with potassium permanganate from the working solution B for 10 min. After washing with distilled water, the cells were stained with Fluoro- Jade C from the working solution C for 10 min. After the washing, the cells were permeabilizated with xylene and examined with a fluorescent microscope (Nikon Instruments Co., Ltd., Tokyo, Japan). The result was expressed in the number of FJC-stained cells in 6 randomly selected non-overlapping fields under high magnification (×200). Five plates had to be counted for each group.

**1.8. Cell Counting Kit-8 (CCK-8) kit**

The cell viability was measured by a CCK-8 kit (Beyotime Biotechnology, Shanghai, China), according to the instructions provided by the manufacturer. Absorbance at a 450-nm wavelength was detected using a microplate reader. Cell viability was calculated using the formula of the survival rate: [(As – Ab)/ (Ac – Ab)] × 100%. Meanwhile, the inhibition rate was calculated using the formula [(Ac – As)/ (Ac – Ab)] × 100%. As: Autophagy intervention group, Ac: OGD group, Ab: Sham group.

**1.9. Statistical analysis**

All the data in this study were subjected to one-way ANOVA or the t-test for statistical differences by SPSS 24.0, and values of *P* < 0.05 were considered statistically significant. The statistical analyses were expressed as a mean ± SEM. Western blot strips were analyzed using Image J, bar statistical histograms were drawn using GraphPad Prism 9, and immunofluorescence graphs were processed using Adobe Photoshop CC 2022.

## RESULT

**2.1. The condition of autophagy predominantly occurs in neurons but barely so in microglia**

**Fig. 1:**
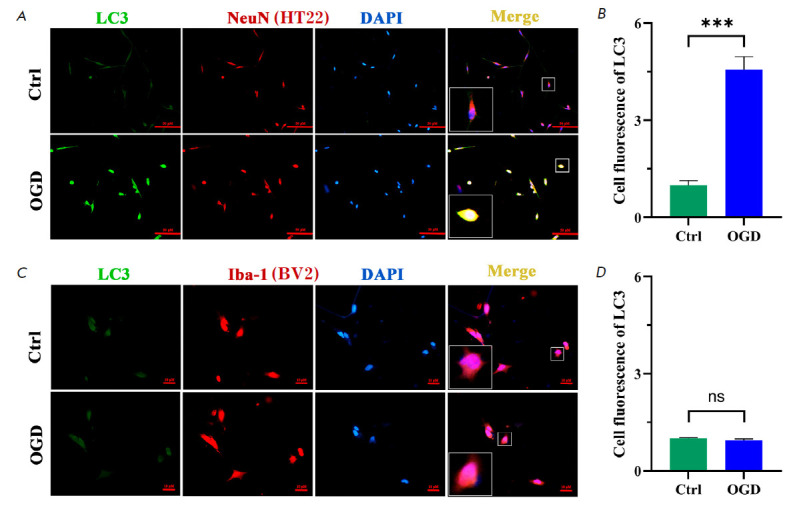
Immunofluorescence was performed to identify the co-culture condition g that meets the requirement that autophagy is mostly induced in HT22 cells but barely in BV2 upon OGD. By Screening, 1.5 h of OGD followed by 2 h of reoxygenation was recognized to meet the requirement that autophagy mainly occurs in neurons but seldom in microglia. (*A*, *C*) Immunofluorescence images of LC3 (green) / NeuN (red), and Iba-1 (red) / DAPI (blue) colocalization. (*B*, *D*) Cell fluorescence of LC3. Bar: 50 μm, *n *= 6. ***p < 0.001, ns

An OGD co-culture model of HT22 neurons with BV2 microglia was prepared. In order to establish the correlation between neuronal autophagy and a microglial inflammation, a culture condition was created firstly so as to meet the requirement that autophagy is mostly induced in HT22, while scarcely so in BV2 cells. Double immunofluorescence demonstrated that 1.5 h of OGD, followed by 2 h of reoxygenation, was the appropriate culture condition in which autophagy is predominantly induced in HT22 cells but barely so in BV2 (*Fig. 1A-D*).

**2.2. OGD-induced neuronal autophagy decreased its CX3CL1 expression**

**Fig. 2:**
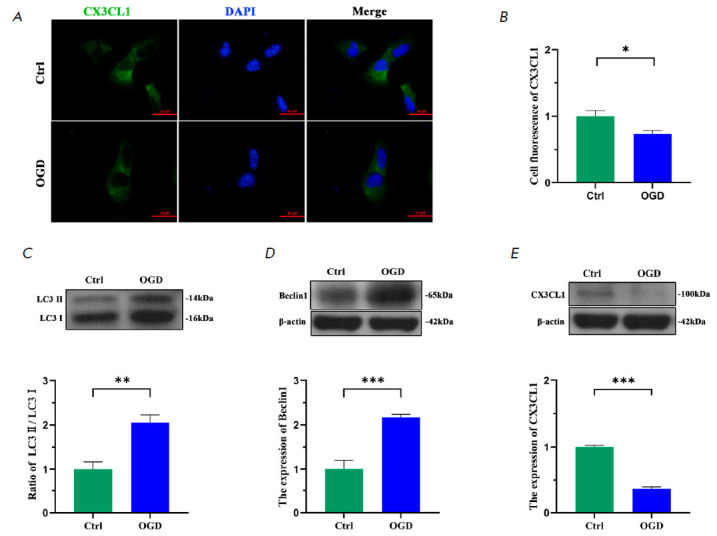
OGD-induced autophagy in HT22 led to decreased CX3CL1 expression. (*A*) Immunofluorescence images of CX3CL1 (green) / DAPI (blue) localization. (*B*) Cell fluorescence of CX3CL1 (green) from image. (*C*–*E*) Western blot images of LC3, Beclin1, CX3CL1, and β-actin expression. Quantitative analysis of the immunoblotted proteins by Image J. Bar: 50 μm, *n *= 6. *p  < 0.05, **p  < 0.01, ***p < 0.001

Autophagy was for the most part induced in HT22 neurons 2 h after OGD, as mentioned above. Thus, the variation in CX3CL1 expression could be directly observed in the HT22 neurons that had suffered from autophagy. Western blot showed that the ratio of LC3-II/LC3-I and the Beclin1 expression were prominently high in the co-cultured cells of HT22 with BV2 (*Fig. 2C,D*), whereas the CX3CL1 expression was conversely low (*Fig. 2E*) in the OGD group compared with those in the control group. Furthermore, double immunofluorescence demonstrated that OGD significantly elevated the autophagy level in the HT22 cells. However, the CX3CL1 expression was contrarily decreased in the OGD HT22 neurons (*Fig. 2A,B*). These results seem to indicate that neuronal autophagy down regulates CX3CL1 expression.

**2.3. Attenuating HT22 autophagy suppresses BV2 inflammatory activation**

**Fig. 3:**
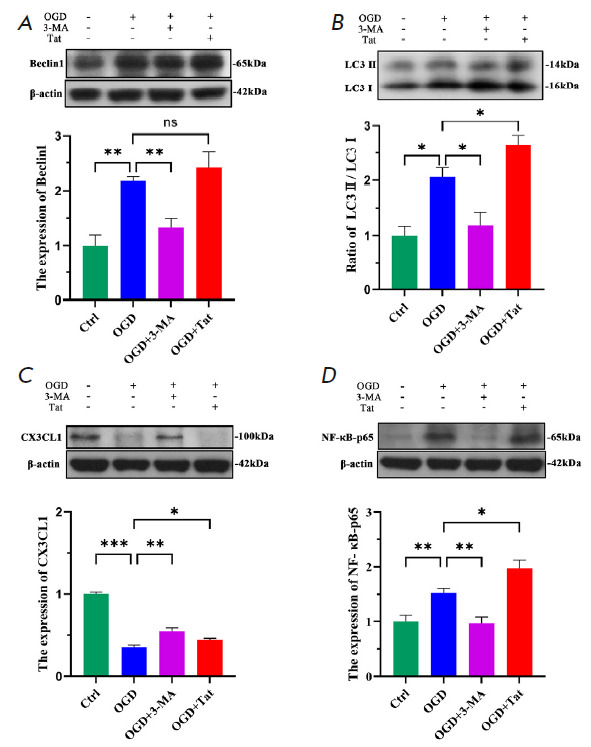
Inhibiting HT22 autophagy suppressed BV2 inflammatory activation. (*A*–*D*) Western blot images of LC3, Beclin1, CX3CL1, NF-κB-p65, and β-actin expression. Quantitative analysis of the immunoblotted proteins by Image J. *n *= 6. *p  < 0.05, **p  < 0.01, ***p < 0.001, ns

**Fig. 4:**
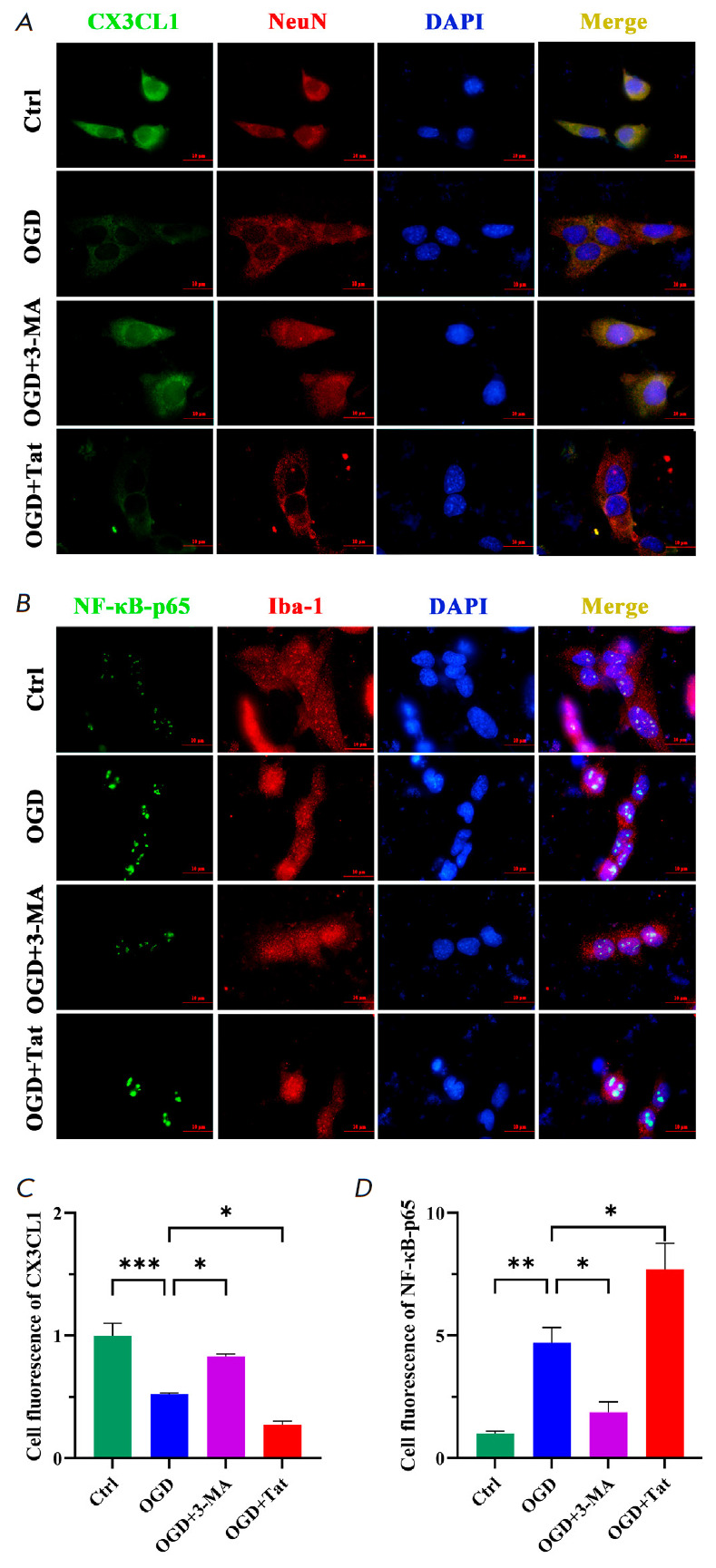
Reducing HT22 autophagy suppressed BV2 inflammatory activation. (*A*, *B*) Immunofluorescence images of CX3CL1 (green) / NeuN (red), NF-κB-p65 (green) / Iba-1 (red), and DAPI (blue). (*C*, *D*) Cell fluorescence of CX3CL1 and NF-κB-p65 (green). Bar: 50 μm, *n *= 6. *p < 0.05, **p  < 0.01, ***p  < 0.001

The OGD-elevated autophagy in HT22 cells resulted in decreased CX3CL1 expression, which likely weakened the efficacy of their suppressive action on the microglial inflammatory response, due to a disruption of the CX3CL1–CX3CR1 cross-talk. We, therefore, inquired whether the BV2-mediated inflammation could be abated by increasing the CX3CL1 expression via the attenuation of autophagy in HT22 cells. To alter the autophagy level, the autophagy inducer Tat-Beclin1 and its inhibitor 3-MA, respectively, were added into the co-culture medium upon OGD. Western blot showed that both the Beclin1 expression and the ratio LC3-II/LC3-I could be effectively altered by the autophagic agents (*Fig. 3A,B*). The promoted autophagy further decreased CX3CL1 expression in the OGD+Tat group, compared with that in the OGD group (*Fig. 3C*). By contrast, decreasing autophagy increased the CX3CL1 expression in our example (*Fig. 3C*). Meanwhile, western blot demonstrated that 15 μM of Tat-Beclin1 greatly boosts autophagy in HT22 neurons (*Fig. 3A,B*). Moreover, Tat-Beclin1-promoted HT22 autophagy markedly reduced its CX3CL1 expression (*Fig. 4C*). Conversely, 3-MA-inhibited autophagy greatly increased its CX3CL1 expression in the OGD+3-MA group (*Fig. 3C*), compared with that in the OGD group. We further investigated the effect of the altered CX3CL1 expression in the HT22 neurons on microglial inflammatory signaling. The result indicates (*Fig. 4A-D*) that the inflammatory signaling of NF-κB-p65 was prominently reinforced by the autophagy-decreased CX3CL1 (*Fig. 4C,D*). Contrarily, the increased CX3CL1 expression through autophagy inhibition proved effective in mitigating the microglial activation.

**2.4. Inhibiting neuronal autophagy repressed the microglial inflammatory response**

**Fig. 5:**
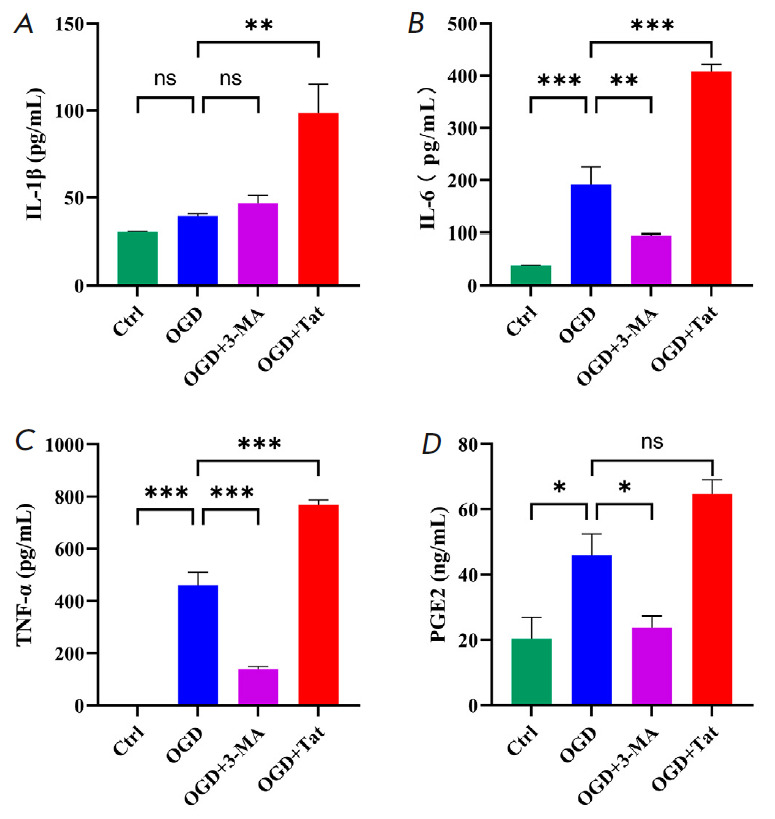
Attenuation of autophagy in HT22 cells repressed the BV2-mediated inflammatory response. ELISA analysis of inflammatory factors including IL-1β (*A*), IL-6 (*B*), TNF-α (*C*), and PGE2 (*D*) converted from the standard curve by measurement of the OD_450_nm value. *n *= 6. *p  < 0.05, **p  < 0.01, ***p  < 0.001, ns

To explore whether the microglial inflammatory response could be suppressed by inhibiting neuronal autophagy, the presence of the inflammatory factors IL-6, IL-1β, TNF-α, and PGE2 was assessed by measuring their concentrations in the OGD co-culture medium. The results (*Fig. 5A-D*) showed that Tat- Beclin1-elevated autophagy in HT22 cells noticeably aggravated the OGD-induced microglial inflammatory response, as was reflected by increased concentrations of IL-6, IL-1β, TNF-α, and PGE2. By contrast, inhibiting autophagy in HT22 cells proved effective in suppressing the inflammation in the OGD+3-MA group, compared with that in the OGD+Tat group or OGD group.

**2.5. Autophagy inhibition-suppressed microglial inflammation benefited neuron survival**

**Fig. 6:**
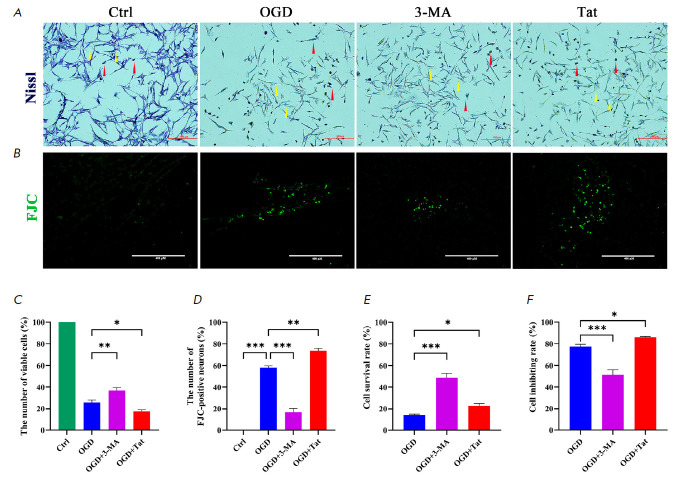
Autophagy inhibition-suppressed microglial inflammation alleviated the neuronal injury after OGD. (*A*) Nissl staining images of pyknotic neurons (red arrows) and viable neurons (yellow arrows). (*B*) FJC staining images of degenerative neurons. (*C*) The number of FJC-positive cells was statistically analyzed. (*D*) The number of viable cells was statistically analyzed. (*E*) Statistical analysis of the cell survival rate detected by the CCK-8 kit. *n *= 6. *p < 0.05, **p < 0.01, ***p < 0.001

To determine whether the CX3CL1-suppressed microglial inflammation could alleviate the OGDinduced neuronal injury, with the CCK-8 kit, Nissl staining and FJC staining were performed to evaluate cell viability, neuron survival, and cell death, respectively. The results showed that cell viability (*Fig. 6A,C*) and neuron survival (*Fig. 6E,F*) were significantly improved, while the cell death rate was correspondingly decreased in the OGD+3-MA group, compared with those in the OGD+Tat group, or in the OGD group. Conversely, the OGD-induced neuronal injury was further aggravated in the OGD+Tat group (*Fig. 6B,D*), compared with that in the OGD group.

## DISCUSSION

Ischemic stroke caused by cerebrovascular occlusion is a fatal disease that threatens human beings [17]. The pathological mechanism underlying ischemic stroke has been extensively investigated in recent years, and yet the recombinant tissue plasminogen activator (rtPA) remains the only approved agent for stroke treatment [18]. Its clinical application can reduce the likelihood of disability by 25%. However, the therapeutic efficacy of rtPA rapidly drops past the 4.5 h that follow an ischemic stroke [19]. Besides, rtPA administration frequently increases the risk of a hemorrhage, which, as we know, aggravates brain injury [19]. Thrombectomy is another efficacious way to remove an infarction, but it may lead to more serious damage to the brain than the cerebral ischemia itself, because of the ischemia/reperfusion injury induced by the instantaneous complete blood resupply [20]. The neurons at the ischemic core rapidly die within several minutes after an ischemic stroke, but the cell death at the penumbra (the peripheral area around the core) lags, due to the milder ischemia by the blood supply with the arterial collateral anastomoses [21]. Mounting evidence points to the fact that neurons that have suffered from autophagy can be rescued back to life by modulation targeting at autophagic/lysosomal signaling [22]. However, neurons and glial cells coexist within penumbra tissues. Thus, this study particularly concerned itself with whether the fate of autophagic neurons is regulated by microglia, using a co-culture of neurons with microglia *in vitro*. This study might provide more clues as to how to improve stroke treatment.

Microglia are native immune cells that are responsible for neuroinflammation and are prominently activated by cerebral ischemia to maintain cellular homeostasis [23]. A modest microglial inflammation benefits neuroprotection, while an amplified immune response leads to neurological injury. CX3CL1, a chemokine anchored to the membranes on neurons, is efficacious in suppressing a microglial inflammation by binding to its receptor CX3CR1 expressed on microglia [6]. Under normal conditions, the microglial inflammatory response can be limited by neurons through the CX3CL1–CX3CR1 signaling pathway [24]. Studies have shown that neuronal autophagy at the penumbra is excessively activated, resulting in aggravated ischemic brain damage [25]. Furthermore, our previous study [16] established that the CX3CL1 expression was significantly reduced on autophagic neurons. We, therefore, looked into whether this reduced CX3CL1 on neurons weakened its suppressive effects on the microglial inflammatory response, resulting in the worsened neurological injury after an ischemic stroke. Based on the CX3CL1-CX3CR1 regulative mechanism, the correlation between neuronal autophagy and the microglial inflammation was investigated using an OGD co-culture of HT22 neurons with BV2 microglia.

Our study demonstrated that 1.5 h of OGD followed by 2 h of reoxygenation was the ideal culture condition under which autophagy was mostly induced in HT22 cells but barely in BV2 (*Fig. 1A,B*). The same study also indicated that neurons were more susceptible to ischemia than microglia, similarly to what was reported in [16]. CX3CL1 was uniquely expressed on neurons. We wondered whether its expression was altered in autophagic neurons. The results demonstrated that the significantly elevated autophagic activity was coupled with a markedly reduced CX3CL1 expression, suggesting that CX3CL1 expression is negatively regulated by autophagy in OGD HT22 neurons. Studies have indicated that a microglial inflammation could be suppressed by neurons through CX3CL1–CX3CR1 signaling [25]. Therefore, we discussed whether the microglial inflammatory injury was aggravated by the down-regulated CX3CL1 expression in OGD HT22 cells. Under OGD condition, the co-cultured cells were treated with the autophagy inhibitor 3-MA and the inducer Tat-Beclin1, respectively. The results showed that attenuation of HT22 autophagy significantly restored CX3CL1 expression (*Fig. 3C*). Consequently, the microglial inflammatory signaling of NF-κB-p65 was greatly suppressed (*Fig. 3D*). Meanwhile, the inflammatory factors of IL-6, IL-1β, TNF-α, and PGE2 were also attenuated (*Fig. 5*). By contrast, promoting HT22 autophagy further reduced its CX3CL1 expression and, in turn, exacerbated the inflammatory response. Moreover, the neuronal autophagy-worsened microglial inflammation led to increased death amongst HT22 cells (*Fig. 6B*). Conversely, down-regulation of autophagy alleviated the inflammatory injury and subsequently promoted neuron survival in OGD HTT cells (*Fig. 6A*). Our data collectively suggest that neuronal autophagy aggravated the microglial inflammatory injury by reducing its CX3CL1, due to the disruption in CX3CL1–CX3CR1 communication that took place after ischemia. Contrarily, promotion of CX3CL1 on neurons by attenuating autophagy could have enhanced its suppressive effects on the microglial inflammatory response and, thereby, alleviate the ischemic injury in the neurons.

In summary, the main purpose of our study was to investigate the correlation between neuronal autophagy and microglial inflammation in a co-culture of neurons with microglia, based on the suppressive impact of neurons on the microglial inflammatory response through CX3CL1–CX3CR1 signaling. The results showed that the OGD-induced neuronal autophagy significantly decreases its CX3CL1 expression, which consequently exacerbates the microglial inflammatory response and neurological injury. Furthermore, promoting neuronal autophagy upon OGD further lessens its CX3CL1 expression and, in turn, worsens the microglial inflammation. Conversely, inhibiting autophagy effectively alleviates the microglial inflammatory injury by up-regulating CX3CL1 expression and, thereby, improving neuronal survival. Our data suggest that inhibiting neuronal autophagy might be a reliable way to alleviate the microglial inflammatory injury after an ischemic stroke.
